# Genotoxic Bystander Signals from Irradiated Human Mesenchymal Stromal Cells Mainly Localize in the 10–100 kDa Fraction of Conditioned Medium

**DOI:** 10.3390/cells10040827

**Published:** 2021-04-07

**Authors:** Vanessa Kohl, Alice Fabarius, Oliver Drews, Miriam Bierbaum, Ahmed Jawhar, Ali Darwich, Christel Weiss, Johanna Flach, Susanne Brendel, Helga Kleiner, Wolfgang Seifarth, Wolf-Karsten Hofmann, Henning D. Popp

**Affiliations:** 1 Department of Hematology and Oncology, Medical Faculty Mannheim, Heidelberg University, 68167 Mannheim, Germany; Vanessa.Kohl@medma.uni-heidelberg.de (V.K.); Alice.Fabarius@medma.uni-heidelberg.de (A.F.); Johanna.Flach@medma.uni-heidelberg.de (J.F.); Susanne.Brendel@medma.uni-heidelberg.de (S.B.); Helga.Kleiner@medma.uni-heidelberg.de (H.K.); Wolfgang.Seifarth@medma.uni-heidelberg.de (W.S.); w.k.hofmann@medma.uni-heidelberg.de (W.-K.H.); 2 Department of Clinical Chemistry, University Medical Center Mannheim, 68167 Mannheim, Germany; Oliver.Drews@umm.de; 3 Department of Radiation Oncology, Medical Faculty Mannheim, Heidelberg University, 68167 Mannheim, Germany; Miriam.Bierbaum@medma.uni-heidelberg.de; 4 Department of Orthopedics and Trauma Surgery, Medical Faculty Mannheim, Heidelberg University, 68167 Mannheim, Germany; Ahmed.Jawhar@medma.uni-heidelberg.de (A.J.); alidarwich@mail.com (A.D.); 5 Department of Medical Statistics and Biomathematics, Medical Faculty Mannheim, Heidelberg University, 68167 Mannheim, Germany; Christel.Weiss@medma.uni-heidelberg.de

**Keywords:** bystander signals, radiation-induced bystander effects, mesenchymal stromal cells, CD34+ cells, leukemia

## Abstract

Genotoxic bystander signals released from irradiated human mesenchymal stromal cells (MSC) may induce radiation-induced bystander effects (RIBEs) in human hematopoietic stem and progenitor cells (HSPC), potentially causing leukemic transformation. Although the source of bystander signals is evident, the identification and characterization of these signals is challenging. Here, RIBEs were analyzed in human CD34+ cells cultured in distinct molecular size fractions of medium, conditioned by 2 Gy irradiated human MSC. Specifically, γH2AX foci (as a marker of DNA double-strand breaks) and chromosomal instability were evaluated in CD34+ cells grown in approximate (I) < 10 kDa, (II) 10–100 kDa and (III) > 100 kDa fractions of MSC conditioned medium and un-/fractionated control medium, respectively. Hitherto, significantly increased numbers of γH2AX foci (*p* = 0.0286) and aberrant metaphases (*p* = 0.0022) were detected in CD34+ cells grown in the (II) 10–100 kDa fraction (0.67 ± 0.10 γH2AX foci per CD34+ cell ∨ 3.8 ± 0.3 aberrant metaphases per CD34+ cell sample; mean ± SEM) when compared to (I) < 10 kDa (0.19 ± 0.01 ∨ 0.3 ± 0.2) or (III) > 100 kDa fractions (0.23 ± 0.04 ∨ 0.4 ± 0.4) or un-/fractionated control medium (0.12 ± 0.01 ∨ 0.1 ± 0.1). Furthermore, RIBEs disappeared after heat inactivation of medium at 75 °C. Taken together, our data suggest that RIBEs are mainly mediated by the heat-sensitive (II) 10–100 kDa fraction of MSC conditioned medium. We postulate proteins as RIBE mediators and in-depth proteome analyses to identify key bystander signals, which define targets for the development of next-generation anti-leukemic drugs.

## 1. Introduction

Genotoxic bystander signals released from irradiated human mesenchymal stromal cells (MSC) may induce radiation-induced bystander effects (RIBEs) in non-irradiated human hematopoietic stem and progenitor cells (HSPC) potentially initiating myeloid neo-plasms (MN). In the 2016 WHO classification, MN that arise after irradiation therapy are referred to as therapy-related MN (t-MN) [1]. As t-MN are characterized by high-risk genetic alterations [2,3] and a particularly worse prognosis [4,5], anti-leukemic therapies are urgently needed.

Generally, RIBEs describe ‘out-of-field’ effects of irradiation in non-irradiated cells that are comparable to effects in irradiated cells. RIBEs may emerge as DNA damage (e.g., increased γH2AX foci, gene mutations, chromosomal aberrations, micronuclei), cell death (e.g., apoptosis, necrosis), and induction of cell survival mechanisms (e.g., adaptive response, DNA repair) [6,7,8,9]. Bystander signals are assumed to be initiated in irradiated cells by calcium fluxes [10] and mitochondrial metabolites [11,12,13]. Afterwards, small molecules like nitric oxide (NO) [14] and reactive oxygen species (ROS) [15] may be transmitted from irradiated cells to non-irradiated bystander cells. Next, regulators, such as nuclear factor-kappa B (NF-kappa B) [13] and transforming growth factor beta-1 (TGFbeta-1) [16,17], may be released by exocytosis. In addition, a role of gap junctions in intercellular transmission has been described [18,19]. Furthermore, cysteine protease cathepsin B is a proven bystander signal [20]. Beyond that, microRNA and mitochondrial DNA might be secreted in exosomes/exosome-like vesicles and contribute to bystander signaling [21,22]. In the last step, NO [23], ROS [23], calcium fluxes [24,25] and distinct factors, such as MAP kinases [25] may be induced in affected bystander cells, thereby potentially initiating malignant transformation.

The analysis of bystander signals is a cutting-edge field in leukemia research. Here, irradiated healthy human MSC and healthy human CD34+ cells from the same donors were investigated in an in vitro model system that enables characterization of genotoxic signaling factors. Specifically, molecular size fractions of MSC conditioned medium of approximate (I) < 10 kDa (corresponding to mediators such as small chemokines, cytokines, survival factors, microRNA), (II) 10–100 kDa (corresponding to mediators such as middle-sized growth factors, cytokines, transcription factors, mtDNA), and (III) > 100 kDa molecular weight (corresponding to large mediators and structures such as exosomes) were used for culture of CD34+ cells of the same donors. Afterwards, RIBE were analyzed in exposed CD34+ cells in terms of DNA damage and chromosomal instability (CIN). The data may provide important information on the fraction of interest in MSC conditioned medium to be analyzed most profitable by in-depth proteome analysis for the identification of key bystander signals, which might contribute to the development of next-generation anti-leukemic drugs.

## 2. Materials and Methods

### 2.1. Collection of Human Femoral Heads

Femoral heads were collected from 7 patients with coxarthrosis (2 female, 5 males, mean age: 71 years, range 52–86 years) undergoing hip replacement.

### 2.2. Isolation of Human MSC

Bones were broken into fragments and incubated for 1 h at 37 °C in phosphate-buffered saline (PBS) supplemented with 1 mg/mL collagenase type I (Thermo Fisher, Waltham, MA, USA). Supernatants were filtered in a cell strainer with 100 µm nylon mesh pores (Greiner Bio-One, Kremsmünster, Austria). Afterwards, bone fragments retained in the cell strainer were transferred into StemMACS MSC Expansion Media XF (Miltenyi Biotec, Bergisch Gladbach, Germany) supplemented with 1% penicillin/streptomycin. Then, adherent MSC were expanded in T175 flasks in a humidified 5% CO_2_ atmosphere at 37 °C and passaged at 80% confluency.

### 2.3. Isolation of Human CD34+ Cells

CD34+ cells were isolated from bone marrow mononuclear cells by Ficoll density gradient centrifugation and magnetic-activated cell sorting using CD34 antibody-conjugated microbeads (Miltenyi Biotec). CD34+ cells were grown in a density of 3 × 10^5^ cells/mL in StemSpan SFEM II medium (Stemcell Technologies, Vancouver, BC, Canada) supplemented with StemSpan Myeloid Expansion supplement (SCF, TPO, G-CSF, GM-CSF) (Stemcell Technologies) and 1% penicillin/streptomycin in a humidified 5% CO_2_ atmosphere at 37 °C.

### 2.4. Preparation of Fractions of MSC Conditioned Medium

MSC were grown in T175 flasks until reaching 80% confluency. MSC were rinsed in PBS and fresh StemSpan SFEM II medium was added. Afterwards, MSC were 2 Gy irradiated by 6 MV X-rays in a Versa HD linear accelerator (Elekta, Stockholm, Sweden), while control MSC were not irradiated. MSC conditioned medium and control medium were obtained from irradiated and non-irradiated MSC, respectively, after 4 h incubation at 37 °C. The collected medium was centrifuged (4000× *g*, 10 min) and supernatants were filtered through 10 kDa molecular weight cut-off (MWCO) ultrafiltration centrifugal filter units (Amicon Ultra, Merck, Darmstadt, Germany) to obtain (I) approximate <10 kDa fractions of MSC conditioned and control medium, respectively. Next, the supernatants above the filter were adjusted with fresh medium to the original volume and filtered through 100 kDa MWCO ultrafiltration centrifugal filter units to obtain (II) approximate 10–100 kDa fractions of MSC conditioned and control medium, respectively. Finally, the supernatants above the filter were adjusted with fresh medium to the original volume and then contained (III) approximate >100 kDa fractions of MSC conditioned and control medium, respectively. The distinct fractions (I)–(III) of MSC conditioned and control medium were stored at −20 °C.

### 2.5. Heat Inactivation of MSC Conditioned and Control Medium

Heat inactivation of RIBE mediators in un-/fractionated MSC conditioned medium and un-/fractionated control medium was performed by incubation at 75 °C for 20 min.

### 2.6. RIBE Analysis

RIBE were analyzed in CD34+ cell samples (#1–6) at day 6 after culture for 3 days in native medium followed by culture for 3 days in un-/fractionated MSC conditioned medium or in un-/fractionated control medium, respectively. Additional experiments with CD34+ cell samples (#5–7) were performed in MSC conditioned medium after heat inactivation.

### 2.7. Immunofluorescence Staining of γH2AX

Immunofluorescence staining of γH2AX was performed in absolute 1 × 10^5^ CD34+ cells using a JBW301 mouse monoclonal anti-γH2AX antibody (1:500) (#05-636, Merck) and an Alexa Fluor 488-conjugated goat anti-mouse secondary antibody (1:500) (#A11001, Thermo Fisher) [26,27]. At least 50 nuclei were analyzed in each sample.

### 2.8. Cytogenetic Analysis

Cytogenetic analysis of G-banded chromosomes was performed in absolute 2 × 10^6^ CD34+ cells according to standard procedures [28]. At least 25 metaphases were analyzed in each sample following the international system for human cytogenetic nomenclature (ISCN) 2016 [29]. Sporadic chromosomal alterations (e.g., chromatid breaks (chtb), chromosome breaks, trisomy) were included in the karyotype (non-clonal events) when detected in at least one metaphase. Because tetraploid/octaploid metaphases were detected at low frequency in CD34+ cells grown in control medium as well, they were only included in karyotypes in case of clonality (tetraploidy and/or octaploidy in two or more metaphases) according to the ISCN 2016.

### 2.9. Statistical Analysis

Statistical analysis was performed with SAS software, release 9.4 (SAS Institute, Cary, NC, USA). For quantitative variables, mean values and standard deviations were calculated. Categorical factors are presented with absolute and relative frequencies. In order to compare more than two groups, Kruskal–Wallis tests were performed. For pairwise group comparisons, exact Wilcoxon two-sample tests were used. In general, test results with *p* < 0.05 were considered as statistically significant.

## 3. Results

### 3.1. DNA Damage in Human CD34+ Cells

**Figure 1:**
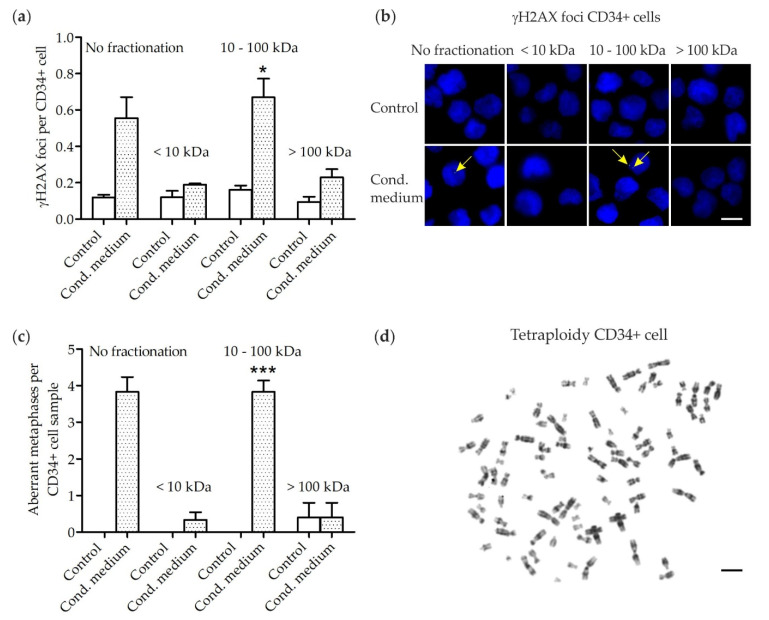
Radiation-induced bystander effects in CD34+ cells grown for 3 days in distinct molecular size fractions of medium conditioned by 2 Gy irradiated mesenchymal stromal cells (MSC) and un-/fractionated control medium. (**a**) γH2AX foci levels in CD34+ cells grown in (I) < 10 kDa, (II) 10–100 kDa and (III) > 100 kDa fractions of MSC conditioned medium and in un-/fractionated control medium. * *p* = 0.0068 (Kruskal-Wallis test) and *p* = 0.0286 (Wilcoxon two-sample test) when compared to numbers of γH2AX foci in CD34+ cells grown in (I) < 10 kDa and (III) > 100 kDa fractions or in un-/fractionated control medium. (**b**) Exemplary images of γH2AX foci (green, Alexa-488) in nuclei (blue, DAPI) of CD34+ cells of patient #2. Scale bar, 5 µm. (**c**) Number of aberrant metaphases in CD34+ cells grown in (I) < 10 kDa, (II) 10–100 kDa and (III) > 100 kDa fractions of MSC conditioned medium and in un-/fractionated control medium. *** *p* = 0.0007 (Kruskal–Wallis test) and *p* = 0.0022 (Wilcoxon two-sample test) when compared to number of aberrant metaphases in CD34+ cells grown in (I) < 10 kDa and (III) > 100 kDa fractions or in un-/fractionated control medium. (**d**) Exemplary tetraploidy of a CD34+ cell grown in the (II) 10–100 kDa fraction of MSC conditioned medium. Scale bar, 10 µm.

γH2AX foci were analyzed in human CD34+ cell samples (4 patients; ∑32 samples) expanded for 3 days in native medium followed by culture for 3 days in un-/fractionated MSC conditioned or un-/fractionated control medium, respectively (Figure 1a,b). Increased numbers of γH2AX foci (general *p* = 0.0068 (Kruskal–Wallis test); pairwise comparison each *p* = 0.0286 (Wilcoxon two-sample test)) were detected in CD34+ cells grown in the (II) 10–100 kDa fraction of MSC conditioned medium (0.67 ± 0.10 γH2AX foci per CD34+ cell; mean ± standard error of mean (SEM)) when compared to numbers of γH2AX foci in CD34+ cells grown in (I) < 10 kDa (0.19 ± 0.01 γH2AX foci per CD34+ cell) and (III) > 100 kDa fractions (0.23 ± 0.04 γH2AX foci per CD34+ cell) of MSC conditioned medium or in un-/fractionated control medium (0.12 ± 0.01 γH2AX foci per CD34+ cell). Since γH2AX foci are a marker of DNA double-strand breaks (DSB), our findings suggest that DNA damage signaling factors mainly localize in the (II) 10–100 kDa fraction of MSC conditioned medium.

### 3.2. Chromosomal Instability in Human CD34+ Cells

Metaphases were analyzed in human CD34+ cell samples (patients #1–6; ∑46 samples) expanded for 3 days in native medium followed by culture for 3 days in un-/fractionated MSC conditioned or un-/fractionated control medium, respectively (Figure 1c,d, Table 1). Increased numbers of aberrant metaphases (general *p* = 0.0007 (Kruskal–Wallis test); pairwise comparison each *p* = 0.0022 (Wilcoxon two-sample test)) were detected in CD34+ cells grown in the (I) 10–100 kDa fraction of MSC conditioned medium (3.8 ± 0.3 aberrant metaphases per CD34+ cell sample; mean ± SEM) when compared to numbers of aberrant metaphases in CD34+ cells grown in (II) < 10 kDa (0.3 ± 0.2 aberrant metaphases per CD34+ cell sample) and (III) > 100 kDa fractions (0.4 ± 0.4 aberrant metaphases per CD34+ cell sample) of MSC conditioned medium or in un-/fractionated control medium (0.1 ± 0.1 aberrant metaphases per CD34+ cell sample). More precisely, distinct chromatid breaks (chtb), e.g., chtb(5q) and chtb(7q) as well as aneuploidies, e.g., tetraploidies and octaploidies, were observed in CD34+ cells grown in the (II) 10–100 kDa fraction of MSC conditioned medium. In addition, distinct chtb, e.g., chtb(2), chtb(9) and chtb(11) as well as aneuploidies, e.g., tetraploidies and octaploidies, were observed in CD34+ cells grown in unfractionated MSC conditioned medium. It has to be noted, that loss of chromosome Y in sample #5 is a common finding in elderly men occurring at a frequency of 5–10% [30,31]. Further, few chromosomal aberrations, e.g., chtb(14q) and aneuploidies, e.g., tetraploidies, were detected at very low frequencies in (I) < 10 kDa and (III) > 100 kDa fractions of MSC conditioned medium, which might arise sporadically or due to limitations in the accuracy of the filtration process.

Finally, heat inactivation of unfractionated MSC conditioned medium and unfractionated control medium (patients #5–7; ∑6 samples) resulted in increased doubling times of CD34+ cells (Table 2). While proliferation of CD34+ cells in sample #5 was reduced, the proliferation of CD34+ cells in samples #6 and #7 was almost regular. All metaphases in CD34+ cells in samples #5 and #6 displayed a normal karyotype when grown in heat-inactivated MSC conditioned medium or control medium. Notably, two tetraploidies were observed in CD34+ cells in sample #7 when grown in heat-inactivated MSC conditioned medium or control medium with an additional chtb(4q) occurring in the same sample when grown in heat-inactivated conditioned medium. While tetraploidies occur frequently in CD34+ cells grown in MSC conditioned medium, they occur sporadically at low frequencies in healthy CD34+ cells and might be even more frequent in aging CD34+ cells of the elderly. Further, the reduced proliferation especially in sample #5 could lead to a lowered number of irregular karyotypes due to cell cycle arrests. Nonetheless, the disappearance of RIBEs in the whole in CD34+ cells grown in heat-inactivated MSC conditioned medium suggests heat-sensitive structures as critical bystander signals.

## 4. Discussion

Genotoxic bystander signals released from irradiated human MSC may induce DNA damage and CIN in human HSPC potentially initiating MN. While increased DNA damage and CIN are readily inducible in human CD34+ cells by exposure to MSC conditioned medium, the genotoxic bystander signals in MSC conditioned medium remain largely uncharacterized yet. Therefore, our study was designed to investigate the molecular features of bystander signals in terms of molecular weight and potential protein characteristics. For this purpose, approximate (I) < 10 kDa, (II) 10–100 kDa and (III) > 100 kDa fractions of MSC conditioned medium were first generated and then unfractionated MSC conditioned medium was heat-inactivated for co-culture experiments in healthy human CD34+ cells of the same donors.

Immunofluorescence microscopy of γH2AX foci, which are a marker of DSB, is widely used in RIBE analysis [32,33,34]. Increased numbers of γH2AX foci were detected in CD34+ cells grown in the (II) 10–100 kDa fraction of MSC conditioned medium when compared to low numbers of γH2AX foci in CD34+ cells grown in (I) < 10 kDa and (III) > 100 kDa fractions of MSC conditioned medium or in un-/fractionated control medium. Our data are in line with similarly increased numbers of chtb detected in CD34+ cells grown in the (II) 10–100 kDa fraction of MSC conditioned medium. Importantly, chtb may activate oncogenes or inactivate tumor suppressor genes, thus providing a potential mechanistic link to the initiation of MN.

Cytogenetic analysis is a suitable method for analysis of RIBE and has been applied in mouse HSPC [35,36]. Increased numbers of aberrant metaphases were observed in CD34+ cells grown in the (II) 10–100 kDa fraction of MSC conditioned medium when compared to low numbers of aberrant metaphases in CD34+ cells grown in (I) < 10 kDa and (III) > 100 kDa fractions of MSC conditioned medium or in un-/fractionated control medium. In particular, the number of tetraploidies was increased in the (II) 10–100 kDa fraction of MSC conditioned medium. Generally, tetraploidies may occur by chromosomal non-disjunction during mitosis or cytokinesis failure [37]. Further, tetrapolidies are found in about 1% of AML but 13% of t-AML cases [38]. Hence, our finding of increased tetraploidies in CD34+ cells grown in the (II) 10–100 kDa fraction of MSC conditioned medium suggests a mechanistic link to the initiation of MN. Although tetraploidies occurred at very low frequency in CD34+ cells grown in control medium, this result is not contradictory to our interpretations but indicates that tetraploidies may randomly occur in vitro during the proliferation process itself. Furthermore, the detection of a clonal tetraploidy in CD34+ cells obtained from an 86-year-old female points to a possible link between tetraploidies and aging [37].

Heat inactivation of unfractionated MSC conditioned medium rescued exposed CD34+ cells from generating excessive chromosomal aberrations. Thus, RIBE mediators have a temperature-sensitive structure supporting the notion that the three-dimensional conformation of macromolecules, such as the native tertiary structure in proteins, confers specifically to the genotoxic effects in the (II) 10–100 kDa fraction of MSC conditioned medium instead of the sheer presence of mediating macromolecules. Our data are in accordance with the results in previous studies demonstrating that heat inactivation of conditioned medium reconstituted cloning efficiencies and cell survival in exposed keratinocytes and chondrocytes, respectively [39,40].

Our study may raise the question for the impact of molecules such as ROS and NO as potential RIBE mediators in the 10–100 kDa fraction of MSC conditioned medium. Considering that ROS and NO are rather short-lived mediator molecules, there might be no major impact of MSC released ROS and NO on detected RIBEs in CD34+ cells in our experiments. More likely, hitherto unknown mediators with a longer half-life may increase ROS and NO in exposed CD34+ cells grown in MSC conditioned medium. Furthermore, large exosomes can be excluded from playing a critical role as vehicles for RIBE mediators in the MSC conditioned medium. In addition, it is important to note that the release of bystander signals and the induction of RIBEs is tissue- and dose-specific thereby following a certain kinetic [41]. While molecules or ions may pass membranes in irradiated MSC by diffusion, gap junctions or ion channels in milliseconds, the synthesis and secretion of proteins by exocytosis and exosomes may take minutes to hours. Subsequently, DNA damage in CD34+ cells may occur in minutes [41], while the formation of complex cytogenetic aberrations during cell divisions requires hours to days. Hence, the time intervals of 4 h post-irradiation for generating MSC conditioned medium and the analysis of RIBEs in CD34+ cells 3 days after exposure to MSC conditioned medium are specific but might be appropriate for analyzing bystander signals and their potential role in MN initiation in vitro.

Finally, our work may have practical importance and contribute to the development of future clinical applications. We first suggest in-depth proteome analysis of the 10–100 kDa fraction of MSC conditioned medium for the identification of key bystander signals. Second, bystander signals should be validated for their oncogenic potential, for example by exposing healthy CD34+ cells to medium containing bystander signal-like recombinant proteins followed by analysis of genetic alterations and potential induction of leukemic clones. Third, monoclonal antibodies against specific bystander signals could be developed, that might then be applied as anti-leukemic prophylaxis after irradiation in pre-clinical studies and, finally, clinical trials.

## 5. Conclusions

In conclusion, our data demonstrate that substantial genotoxic bystander signals mainly localize in the (II) 10–100 kDa fraction of MSC conditioned medium and that these signals are heat-sensitive. Based on these biochemical properties, we postulate proteins as RIBE mediators, which should be further analyzed by an in-depth proteome analysis of the corresponding fraction. Ultimately, it has the potential to uncover the identity of key bystander signals, which is fundamental for the development of next-generation anti-leukemic drugs.

## Figures and Tables

**Table 1:** Cytogenetics in CD34+ cells grown for 3 days in un-/fractionated medium conditioned by 2 Gy irradiated mesenchymal stromal cells. CM, conditioned medium; NA, not assessed; Pt, patient; [number] = number of metaphases.

Pt	Age/	Cytogenetics CD34+ Cells		Cytogenetics CD34+ Cells		Cytogenetics CD34+ Cells		Cytogenetics CD34+ Cells	
	Sex	No Fractionation		<10 kDa		10–100 kDa		>100 kDa	
		Control	CM	Control	CM	Control	CM	Control	CM
#1	84/♀	46,XX[25]	46,XX[20] 53,XX,+1,+2,+5,+6,+14,+21,+22[1] 92,XXXX[4]	46,XX[20]	46,XX[25]	46,XX[25]	46,XX[22] 92,XXXX[3]	NA	NA
#2	65/♂	46,XY[25]	46,XY[20] 92,XXXX[1] 184,XXXXYYYY,chtb(11)(q23)[1] 46,XY,dup(13q)[1] 47,XY,+21,chtb(11)(p12)[1] 46,XY,chtb(9)(12)[1]	46,XY[25]	46,XY[22]	46,XY[25]	46,XY[21] 92,XXXX[2] 69,XXY[1] 47,XY,+3[1]	46,XY[25]	46,XY[25]
#3	62/♂	46,XY[25]	46,XY[22] 92,XXYY[3]	46,XY[25]	46,XY[25]	46,XY[25]	46,XY[20] 92,XXYY[3] 46,XY,chtb(5)(q33)[1] 46,XY,+f[1]	46,XY[25]	46,XY[25]
#4	62/♂	46,XY[25]	46,XY[21] 92,XXYY[3] 92,XXYY,chtb(2p)[1]	46,XY[25]	46,XY[23] 46,XY,chtb(14q)[1]	46,XY[25]	46,XY[22] 92,XXYY[1] 184,XXXXYYYY[1] 46,XY,chtb(7p)[1]	46,XY[23] 184,XXXXYYYY[2]	46,XY[25]
#5	85/♂	46,XY[13] 45,X,-Y[12]	46,XY[10] 45,X,-Y[12] 90,XX,-Y,-Y[1] 92,XXYY[1] 184,XXXXYYYY[1]	46,XY[5] 45,X,-Y[20]	46,XY[7] 45,X,-Y[18]	46,XY[21] 45,X,-Y[4]	46,XY[18] 45,X,-Y[3] 92,XXYY[2] 47,XY,+2[1] 50,XY,+1,+7,+9,+14[1]	46,XY[10] 45,X,-Y[15]	46,XY[13] 45,X,-Y[7] 92,XXYY[1] 90,XX,-Y,-Y[1]
#6	52/♂	46,XY[25]	46,XY[22] 92,XXYY[2] 184,XXXXYYYY[1]	46,XY[25]	46,XY[24] 46,XY,+f[1]	46,XY[21]	46,XY[21] 92,XXYY[3] 184,XXXXYYYY[1]	46,XY[25]	46,XY[25]

**Table 2:** Cytogenetics and cell doubling time in CD34+ cells grown for 3 days in heat-inactivated unfractionated medium conditioned by 2 Gy irradiated mesenchymal stromal cells. CM, conditioned medium; Pt, patient; [number] = number of metaphases.

Pt	Age/	Cytogenetics CD34+ Cells		Cytogenetics CD34+ Cells		Cell Doubling Time (Days)		Cell Doubling Time (Days)	
	Sex	Control	CM	Control	CM	Control	CM	Control	CM
		No Heat Inactivation		+Heat Inactivation		No Heat Inactivation		+Heat Inactivation	
#5	85/♂	46,XY[13] 45,X,-Y[12]	46,XY[10] 45,X,-Y[12] 90,XX,-Y,-Y[1] 92,XXYY[1] 184,XXXXYYYY[1]	46,XY[15]	46,XY[14]	1.0	1.0	1.9	1.5
#6	52/♂	46,XY[25]	46,XY[22] 92,XXYY[2] 184,XXXXYYYY[1]	46,XY[25]	46,XY[24]	1.2	1.0	1.5	1.7
#7	86/♀	46,XX[25]	46,XX[20] 46,XX,chtb(10q)[1] 46,XX,del(19)(p10)[1] 92,XXXX[2] 184,XXXXXXXX[1]	46,XX[23] 92,XXXX[2]	46,XX[22] 92,XXXX[2] 46,XX,chtb(4q)[1]	1.3	1.2	1.8	1.4

## Data Availability

Data are contained within the article.
